# Characteristics and Influencing Factors of Healthcare Utilization in Post-conflict Primary Care Attendees in Northern Sri Lanka

**DOI:** 10.3389/frhs.2021.719617

**Published:** 2022-01-28

**Authors:** Shannon Doherty, Diliniya Stanislaus Sureshkumar, Rasiah Thayakaran, Rajendra Surenthirakumaran

**Affiliations:** ^1^School of Allied Health, Faculty of Health, Education, Medicine and Social Care, Anglia Ruskin University, Cambridge, United Kingdom; ^2^Unit for Social and Community Psychiatry, WHO Collaborating Centre for Mental Health Service Development, Queen Mary University of London, London, United Kingdom; ^3^Institute of Applied Health Research, University of Birmingham, Birmingham, United Kingdom; ^4^Department of Community and Family Medicine, University of Jaffna, Jaffna, Sri Lanka

**Keywords:** primary care, mental health, health service access and utilization, post-conflict, healthcare

## Abstract

Sri Lanka's healthcare systems attempts to provides access to universal healthcare services for all citizens and is designed to be free of out-of-pocket payments. Despite a 30-year civil conflict, natural disasters, and COVID-19, the healthcare system within the country remains robust and strong. However, due to a lack of formalized pathways and centralized record keeping, the pattern of service utilization is still relatively unknown, which raises concerns regarding effective allocation of scarce resources and efficiency of referral pathways. To address this gap in knowledge, part of the parent study (COMGAP-S), consisted of a survey on healthcare service use conducted among adults attending primary care facilities. The results from this quantitative data analysis indicate the majority of people seeking care originate from rural areas, are older (50+), attend divisional hospitals, and report paying fees at point of contact. Our findings indicate a need for more efficient use of healthcare services, creation of referral routes to ensure limited healthcare resources are used effectively. Additionally, further investment in services is needed to ensure Sri Lanka's healthcare system meets the standard of universal healthcare as proposed by the World Health Organization. These findings address a gap in knowledge for national decision-makers within Sri Lanka along with other similar post-conflict healthcare settings across the globe.

## Introduction

The country of Sri Lanka provides a publicly funded, universal healthcare system that is meant to be available to all without restriction (1, 2). Within Sri Lanka's healthcare system, inpatient and outpatient services are available within divisional hospitals, while primary medical care units provide only outpatient care. Primary medical care units and outpatient services at divisional hospitals comprise the “primary care” level of Sri Lanka's healthcare system. Secondary care (surgeons, psychiatrists, ob-gyn consultants) and tertiary care (cardiologists, intensivists) are provided at divisional hospitals, but not primary medical care units (3). Within the primary care system, primary curative care is provided by public hospitals and general practitioners, while primary care preventative care are services provided by healthcare professionals such as public health nurses and midwives (3). As noted by Mendis (3) primary care preventive services have been neglected in Sri Lanka and there is no data available on the number of patients that visit outpatient services nor is there any morbidity patterns to understand why patients attend primary care services. The existence of a universal healthcare program encourages health equity and aligns with the United Nation's Sustainable Development Goals (4). However, the primary care system has been weakened by underinvestment and shortages in medical supplies and human resources creating an inability to provide sufficient levels of continuity of care (2). The emergence of COVID-19 has further impacted the country, which could have long-lasting, negative impacts on healthcare services. Even facing adversities of conflict, natural disaster, internal displacement, and now a pandemic, Sri Lanka's under-five mortality rate is low, and 99% of women (15–49 years) received postnatal care within 2 days of giving birth (4). This indicates that the health system has a strong foundation in child/maternal health already meeting one of the SDGs Goal 3 indicators. Further, life expectancy stands at 73 (m) and 79 (f) years indicating excellent health outcomes despite a relatively low GDP per capita of US $3852 (5–9). Sri Lanka's healthcare system therefore, appears robust and seems to contribute to good health outcomes, particularly if compared to other middle-income countries (1).

However, while the healthcare system in Sri Lanka appears strong, not much is known about factors of healthcare utilization by primary care attendees seeking outpatient and inpatient services. This is in part due to the lack of centralized record keeping within primary care facilities–each patient has their own record book and there is no existing central records system (3). Thus, while access to healthcare facilities is provided, the research on utilization by patients is scant.

In 2017, Pallegedara and Grimm published a paper examining healthcare utilization behavior in Sri Lanka with an emphasis on choices made between private and public healthcare. The authors conducted an analysis using national household survey and district level healthcare supply data and identified significant regional and ethnic differences in healthcare access (1). This paper noted a trend in income associated with private health care utilization as those with more wealth more often seeking out private healthcare, driving demand and decreasing demand for public healthcare services. This in turn may affect access and quality of public healthcare for all Sri Lankans, particularly in rural regions. Further, as pathways through care do not formally exist, or are undocumented, this could mean pathways through care are not being used in effective or cost-efficient ways (8). Of particular interest are questions around who is utilizing services, and the pattern of utilization (what services are being accessed and for what purpose). These unanswered questions make it unclear if pathways through healthcare are efficient and effective. For example, if a patient sees a primary care practitioner at a primary care medical unit and is then referred on to a divisional hospital for specialized care, this journey is not documented in a centralized system (3). This is especially vital to understand as prevalence of non-communicable diseases (NCDs) and mental health issues are rising (10), and such complex issues require clear pathways. These are not just issues within the Sri Lankan post-conflict context, but are common across similar settings across the globe (11–13).

Research within comparable settings note similar concerns in relation to delivery of healthcare in post-conflict settings. Within the postwar setting of Liberia, Lee et al. (11) note the need for better data management to ensure chronic diseases are addressed effectively and the need to bridge the rural health delivery gap. This is similar to work by Nelson et al. (12) which examined the ability of the healthcare system in Serbia to provide for the needs of its citizens. The authors found lack of financial investment, inadequate supplies and equipment to be some of the contributing barriers to development of, and access to, healthcare services. Research conducted by Kentoffio et al. (13) in post-war Liberia which found that despite general success of post-war rehabilitation, rural populations were still lacking access to basic care.

Regarding healthcare financing, research from Wang et al. (14) notes that while Sri Lanka claims a universal healthcare system, out-of-pocket expenditures do occur, and appear to be related to purchase of medications. This kind of expenditure may disproportionately affect poorer households as they often have fewer resources at their disposal to meet the cost (14). While implementation of pharmaceutical policies across the country may help alleviate these costs, this may only apply to publicly funded healthcare facilities and often people buy medicines in private pharmacies (14). Kumar (2) further notes that underinvestment in the healthcare system has resulted in shortages of medical supplies and services, and human resources across the country. This is especially concerning with the rise of NCDs in the country which require access to equitable services and clear referral pathways to ensure continuity of care.

This is supported by work conducted within Sri Lanka by the Ministry of Health, Nutrition and Indigenous Medicine in Northern Province (10). Their report indicates health conditions such as ischemic heart disease and stroke account for 40% of the country's premature deaths. Further, as Sri Lanka's aging population is set to put more pressure on the health system and increase service costs (9). Their report presents findings from a stakeholder meeting and notes primary care facilities are currently underutilized due to a lack of lab tests, basic medicines, and unsuitable hours of service (8, 10). Further, it is recommended that protocols are lacking for treatment guidelines and management of referrals from primary health care to higher levels of care (8, 10).

It is imperative for regional and national healthcare decision-makers to understand healthcare utilization to ensure resources are used efficiently and effectively. While health expenditure per capita has increased in Northern Province quite substantially, the utilization of healthcare resources is still unknown. Further, who is using services, and their pattern of utilization is unknown which is essential to understand if primary health care services are to be used efficiently. Healthcare expenditure should be grounded on a bedrock of evidence around characteristics and pattern of service utilization to be efficient and effective. This could also help ensure continuing health equity and effective financing plans. Further, with the prevalence of mental health disorders, and the increase in NCDs within the country, it is imperative to ensure pathways of care of understood and used effectively (10). This information could then be utilized in similar post conflict settings which often face comparable issues related to effective and efficient allocation and use of healthcare services. It is against this backdrop that this quantitative study was undertaken to address the current gap in knowledge.

This current study sought to address the current gap in knowledge and add to the evidence base for national level healthcare decision-makers in Sri Lanka and other similar post conflict settings across the globe. It is hoped that lessons learned from the Sri Lankan context could be valuable to other similar post-conflict settings. Around the world there has been an increase in conflict and natural disasters, and countries have limited resources to allocate to healthcare. Thus, understanding healthcare utilization within this post-conflict setting could provide valuable information for decision-makers within Sri Lanka and those in similar contexts. This is particularly important due to the COVID-19 pandemic increasing strain on healthcare systems worldwide.

The aim of the current study was to understand patterns of utilization among primary care attendees in Northern Province, Sri Lanka. The main objectives were to (1) explore characteristics of healthcare utilization by conflict-affected, adult primary care attendees, (2) examine health service utilization within primary care facilities that provide outpatient and inpatient care services.

## Materials and Methods

This quantitative data analysis utilizes data from the COMGAP-S study (15). In phase 1 of COMGAP-S an epidemiologic survey was conducted among adults attending primary care facilities across all five districts of Northern Sri Lanka: Jaffna, Mullaitivu, Vavuniya, Kilinochchi, and Mannar. The purpose of the parent survey was to estimate prevalence of mental health disorders and epidemiological associations within a post-conflict population. Sample sizes for the parent study were calculated using a 50% prevalence rate of the most common mental health disorders, anxiety or depression, based on previous studies in the region (15). The primary sampling units were the public primary care facilities within the Northern Province, which included both divisional hospitals and primary care medical units. A list of all public primary care facilities was obtained and compiled by type and 25 facilities were randomly chosen. Clusters within each of the 5 districts of Northern Province were allocated proportionally according to total numbers of internally displaced populations. This was done to account for population displacement within the last stage of the Civil War (2009) as some regions of the Northern Province experienced less displacement but had larger populations. This ensured the parent study had an adequate representation of displacement and conflict severity. Within each facility (cluster), 41 individuals were recruited through systematic selection of every third attendee at the facility registration desk (15). Informed written consent was provided in English, Sinhalese or Tamil (depending on participant language preference). Informed consent was obtained from all participants and participants were free to withdraw from the study at any point during data collection. Effort was taken to ensure participants were interviewed in private spaces within the facilities to protect privacy and ensure confidentiality. All participants were assigned a participant number after signing the informed consent to ensure confidentiality (15).

Data was collected for the parent study using a structured interview which covered demographic information, conflict and displacement experience and a range of mental health screening tools. Data collectors were Sri Lankan research assistants who spoke English, Tamil and Sinhala. Data collectors were trained over a 7-day period by a senior academic in Jaffna, Sri Lanka on how to administer the questionnaire along with how to ethically gain consent. As part of this questionnaire packet, participants were asked about their healthcare service utilization and this paper presents the results from that survey. This quantitative analysis sought to explore the characteristics of inpatient and outpatient care received at primary care level and healthcare utilization. To understand the characteristics of inpatient and outpatient care, a modified version of the Client Service Receipt Inventory (CSRI) was used. This modified measure, titled the Health Service Use questionnaire, had been previously validated in Sri Lanka, translated, back-translated and adapted for local context (15–17). The questionnaire was piloted before implementation within the full COMGAP-S study to ensure validity and cultural appropriateness. To understand possible factors that could influence perception of care, information collected through a structured interview covering socioeconomic background, along with conflict and displacement experiences were analyzed.

Data were downloaded to SPSS version 26.0 from a secure server, and SPSS was used for the secondary data analysis (18). Data were inspected and cleaned by the Principal Investigator as data were uploaded throughout data collection in real time. For the current analysis, to understand the characteristics of inpatient care, the following dependent variables were examined: reason for admission, place of admission, if the admission was overnight or not, and fees paid at point of service. To understand the characteristics of outpatient care, the following dependent variables were examined: reason for visit, type of healthcare professional seen, place healthcare professional was seen, and the main feature of the visit. Associations between characteristics and ethnicity, gender, education, occupation, house location (urban/rural), and combatant/non-combatant status (independent variables) were investigated using Chi square, Fishers Exact test or Monte Carlo methods depending on which test was appropriate. Binary logistic regressions were then run on significant results with both outpatient and inpatient groups. The outpatient binary logistic regression was performed with significant sociodemographic characteristics (age, ethnicity, location of home, education, occupation) and reasons for attending outpatient services along with healthcare provider seen (age, ethnicity, location of home, occupation). The inpatient binary logistic regression was performed with significant sociodemographic characteristics (age, ethnicity, location of home, education, occupation) and reasons for admission to inpatient services and overnight stay (age, education). Dummy coding was used to code the dependent data and explanatory models were based on previous research in the region (15).

## Results

Of the 1,025 participants approached in primary care facilities, a total of 1,019 completed the Health Service Use survey. Over half the participants were older than 50 years (59.2%) and split almost evenly between male (47.5%) and female (52.5%) (Table 1). The majority of participants were of Tamil ethnicity (89.2%) and were living in a rural area (84.2%). The majority of participants were either employed (42.8%) or at home/housewife (40.2%), and most education levels ranged from Grade 1 (30.2%) to Grade 6 (35.9%). Very few participants stated they had participated directly in combat (2.6%), but all participants had been displaced at least once during the Sri Lankan Civil War. Please (see Table 1) for an overview of participant sociodemographic information.

**Table 1:** Participant sociodemographics.

**Participant Sociodemographics**	***N* (%)**
**Gender (*****N*** **= 1,019)**	
Male	484 (47.5)
Female	535 (52.5)
**Age groups (*****N*** **= 1,019)**	
18–34	141 (13.8)
35–49	275 (27.0)
50–64	335 (32.9)
65+	268 (26.3)
**Home location (*****N*** **= 1,017)**	
Rural/village/plantation/coastal	930 (91.4)
Urban/city/town	87 (8.5)
**Occupation (*****N*** **= 1,018)**	
Employed	436 (42.8)
Unemployed/disabled	102 (10.0)
Housewife/At home	410 (40.2)
Student	19 (1.8)
Retired	44 (4.3)
Other	7 (0.9)
**Education (*****N*** **= 1,019)**	
No school education	67 (6.6)
Up to grade 5	308 (30.2)
Grade 6 to O/Ls	366 (35.9)
Passed O/Ls	146 (14.3)
Up to A/Ls	87 (8.5)
University or other	45 (4.5)
**Ethnicity (*****N*** **= 1,019)**	
Tamil	909 (89.2)
Muslim	110 (10.8)
**Marital status (*****N*** **= 1,008)**	
Married	802 (79.6)
Widowed	140 (13.9)
Separated	16 (1.6)
Divorced	2 (0.2)
Never married	48 (4.7)
**Religion (*****N*** **= 1,019)**	
Hindu	681 (66.8)
Islam	113 (11.1)
Christian	220 (21.6)
Other	5 (0.5)

### Outpatient and Reasons for Healthcare Visits

There was a significant association between male gender and OP services sought for injuries [X^2^ (1, *N* = 240) = 54.187, *p* < 0.001] with more men than women seeking treatment (Table 2). A total of 205 (20.1%) participants attended OP services for chronic disease and a significant relationship was found between age groups and visiting OP facilities for chronic disease, [X^2^ (3, *N* = 205) = 55.928, *p* < 0.001]. Participants 50 years and older (80.5%) were more likely to visit OP services for chronic disease (such as diabetes) than those 18–49 years (19.6%). Participants 18–34 were more likely to attend for acute conditions (such as flu) or injuries. The relationship between education levels and OP visits for chronic disease was significant, [X^2^ (6, *N* = 180) = 26.428, *p* < 0.001]. Participants who had completed Grade 1–5 (38.5%) or Grade 6 (34.1%) were more likely to attend OP services than participants who had no formal schooling (7.3%) or who had passed their Grade 6 (7.8%) (Table 2).

**Table 2:** Outpatient characteristics and reason for visit.

	**Injury**	**Infectious disease**	**Acute conditions**	**Sleep problems**	**Chronic disease**	**Other reason**
**Age groups**						
18–34	37 (15.4%)	4 (26.7%)	39 (23.2%)	1 (3.6%)	4 (2%)	68 (12%)
34–49	72 (30%)	1 (6.7%)	55 (32.7%)	8 (28.6%)	36 (17.6%)	157 (27.7%)
50–64	68 (28.3%)	4 (26.7%)	45 (26.8%)	10 (35.7%)	86 (42%)	190 (33.5%)
65+	63 (26.3%)	6 (40%)	29 (17.3%)	9 (32.1%)	79 (38.5%)	152 (26.8%)
Pearson chi-squared	0.330	0.146	**0.001**	0.445	**0.001**	0.288
**Ethnicity**						
Tamil	226 (94.2%)	14 (93.3%)	153 (91.1%)	26 (92.9%)	168 (82%)	510 (89.9%)
Muslim	14 (5.8%)	1 (6.7%)	15 (8.9%)	2 (7.1%)	37 (18%)	57 (10.1%)
Pearson chi-squared	**0.005**	-	0.412	-	**0.001**	0.442
Fisher's exact test	-	1.000	-	0.760	-	-
**Gender**						
Male	164 (68.3%)	8 (53.3%)	71 (42.3%)	13 (46.4%)	87 (42.4%)	243 (42.9%)
Female	76 (31.7%)	7 (46.7%)	97 (57.7%)	15 (53.6%)	118 (57.6%)	324 (57.1%)
Pearson chi-squared	**0.001**	0.654	0.130	0.901	0.098	**0.001**
Fisher's exact test	-	-	-	-	-	-
**Location**						
Rural/village/plantation	214 (89.2%)	13 (86.7%)	142 (84.5%)	19 (67.9%)	186 (91.2%)	532 (93.9%)
Urban/city/town	26 (10.8%)	2 (13.3%)	26 (15.5%)	9 (32.1%)	18 (8.8%)	34 (6.1%)
Fisher's exact test	0.520	0.718	**0.006**	**0.001**	0.978	**0.005**
**Occupation**						
Employed	130 (58.0%)	6 (50%)	71 (43.3%)	10 (38.5%)	70 (36.4%)	225 (41.7%)
Unemployed/off sick/ disabled	27 (12.0%)	1 (8.3%)	13 (8.0%)	2 (7.7%)	30 (15.6%)	64 (11.9%)
Student	5 (2.2%)	-	6 (3.6%)	1 (3.8%)	1 (0.5%)	7 (1.3%)
Housewife/at home	62 (27.8%)	5 (41.7%)	74 (45.1%)	13 (50.0%)	91 (47.5%)	243 (45.1%)
Pearson chi-squared	**0.001**	0.216	0.256	0.623	**0.004**	0.045
**Education**						
Not had school education	14 (6.9%)	1 (11.1%)	7 (5.1%)	2 (7.1%)	15 (8.3%)	45 (9.0%)
From grade 1–grade 5	61 (30.0%)	4 (44.4%)	41 (30.1%)	11 (39.3%)	79 (43.9%)	183 (36.2%)
From grade 6–O/Ls	78 (38.4%)	-	54 (39.8%)	12 (42.9%)	70 (38.9%)	212 (41.9%)
Passed O/Ls	50 (24.7%)	4 (44.4%)	34 (25.0%)	3 (10.7%)	16 (8.9%)	65 (12.9%)
Pearson chi-squared	-	-	-	-	-	-
Fisher's exact test	**0.001**	**0.001**	0.12	0.631	**0.001**	**0.001**

*Bold values are statistically significant results*.

A binary logistic regression (Table 3) indicated a significant association between older age (above 50 years) and outpatients attending for chronic disease [OR, 3.412; 95% CI, 2.31–5.03, *p* < 0.000] and identifying as Tamil ethnicity [OR, 0.430; 95% CI, 0.27–0.67, *p* < 0.0000]. Table 4 shows significant associations were noted between outpatients attending for injury and Tamil ethnicity [OR, 2.161; 95 % CI, 1.20–3.88, *p* < 0.010], being unemployed/student/retired [OR, 0.541; 95% CI, 0.40–0.72], and education levels below O/L [OR, 0.588; CI, 0.43–0.80, *p* < 0.001]. Table 5 illustrates an association between outpatients seen for acute disease and older age [OR, 0.525; 95% CI, 0.37–0.74, *p* < 0.0000], education below O/S [OR, 0.634; 95% CI, 0.44–0.91, *p* < 0.14], and living in a rural/coastal area [OR, 2.332; 95% CI, 1.41–3.84, *p* < 0.001].

**Table 3:** Binary logistic regression showing association of outpatients admitted for chronic disease.

***n* = 1,019**	**Adjusted odds ratio**	***P*-value**	**95.0% CI**	
			**Lower**	**Upper**
**Age**				
Below 50 years 416 (40.8%)	1 (Ref)			
Above 50 years 603 (59.2%)	3.412	**0.000**	2.314	5.032
**Ethnicity**				
Muslim 110 (10.8%)	1 (Ref)			
Tamil 909 (89.2%)	0.430	**0.000**	0.274	0.673
**Job**				
Currently employed 436 (42.8%)	1 (Ref)			
Unemployed/ student/retired 583 (57.2%)	1.466	0.023	1.053	2.040
**Education**				
Above O/L 278 (27.3%)	1 (Ref)			
Below O/L 741 (72.7%)	1.094	0.658	0.734	1.633

*Negelkerke R^2^ = 0.105, Chronic disease; yes = 205, no = 814. Bold values are statistically significant results*.

**Table 4:** Binary logistic regression with outpatients seen for injury.

***n* = 1,019**	**Adj odds ratio**	***P*-value**	**95.0% CI**	
			**Lower**	**Upper**
**Ethnicity**				
Muslim 110 (10.8%)	1 (Ref)			
Tamil 909 (89.2%)	2.161	**0.010**	1.202	3.886
**Employment**				
Currently employed 436 (42.8%)	1 (Ref)			
Unemployed/ student/retired 583 (57.2%)	0.541	**0.000**	0.403	0.727
**Education**				
Above O/L 278 (27.3%)	1 (Ref)			
Below O/L 741 (72.7%)	0.588	**0.001**	0.430	0.806

*Negelkerke R^2^ = 0.053, Injury; yes = 240, no = 779. Bold values are statistically significant results*.

**Table 5:** Binary logistic regression with outpatients seen for acute diseases.

***n* = 1,019**	**Adj odds ratio**	***P*-value**	**95.0% CI**	
			**Lower**	**Upper**
**Age**				
Below 50 years 416 (40.8%)	1 (Ref)			
Above 50 years 603 (59.2%)	0.525	**0.000**	0.370	0.747
**Education**				
Above O/L 278 (27.3%)	1 (Ref)			
Below O/L 741 (72.7%)	0.634	**0.014**	0.440	0.913
**Location of home**				
Urban/city 89 (9.7%)	1 (Ref)			
Rural/coastal area 930 (91.3%)	2.332	**0.001**	1.413	3.849

*Negelkerke R^2^, 0.058, Acute disease; yes = 168, no = 851. Bold values are statistically significant results*.

#### Outpatient Outcome of Visit

Participants in the age group 50–64 (33.6%) were more likely to get an assessment/diagnosis than participants aged 18–34 (10.6%), 35–49 (27.8%) or 65 + (28%) (Table 6). The relation between location and assessment/diagnosis was also significant, (*p* < 0.001). Table 6 also details that three quarters of participants living in a village/rural area (86.5%) were more likely to get an assessment/diagnosis than city/town/urban area (5.5%), or coastal fishing community (7.4%).

**Table 6:** Outcome from outpatient visit.

	**Assessment /Diagnosis**	**Drug prescription**	**Follow up visit**
**Age groups**			
18–34	62 (10.6%)	67 (13.3%)	4 (3.6%)
35–49	163 (27.8%)	145 (28.7%)	18 (16.1%)
50–64	197 (33.6%)	157 (31.1%)	48 (42.9%)
65+	164 (28%)	136 (26.9%)	42 (37.5%)
Pearson chi-squared	**0.005**	0.495	**0.001**
**Ethnicity**			
Tamil	514 (87.7%)	451 (89.3%)	96 (85.7%)
Muslim	72 (12.3%)	54 (10.7%)	16 (14.3%)
Pearson chi- square	0.061	0.971	0.197
**Gender**			
Male	280 (47.8%)	226 (44.8%)	43 (38.4%)
Female	306 (52.2%)	279 (55.2%)	69 (61.6%)
Pearson chi-squared	0.915	0.067	**0.038**
**Location**			
Village/rural area	553 (94.5%)	447 (89.0%)	99 (88.4%)
City/town/urban area	32 (5.5%)	56 (11.0%)	13 (11.6%)
Pearson chi-squared	-	-	-
Fisher's exact test	**0.001**	**0.013**	0.522
**Occupation**			
Employed	255 (46.2%)	209 (42.4%)	37 (35.6%)
Unemployed/off sick/disabled	65 (11.8%)	52 (10.6%)	16 (15.4%)
Student	8 (1.4%)	11 (2.2%)	-
Housewife/at home	224 (40.6%)	221 (44.8%)	51 (49.0%)
Pearson chi-squared	-	-	-
Fisher's exact test	0.306	**0.002**	0.027
**Education**			
Not had school education	47 (8.9%)	32 (7.1%)	9 (9.2%)
From grade 1–grade 5	185 (35.4%)	158 (35.0%)	41 (41.8%)
From grade 6–O/Ls	230 (44.0%)	184 (40.8%)	43 (43.9%)
Passed O/Ls	61 (11.7%)	77 (17.1%)	5 (5.1%)
Pearson chi-squared	-	-	-
Fisher's exact test	**0.001**	**0.001**	**0.001**

*Bold values are statistically significant results*.

Table 6 notes a little under half (49.55%) of participants attended OP services for drug prescriptions. The relation between location and drug prescription was significant, (*p* = 0.013). Over three quarters of participants living in a village/rural area (80.2%) were more likely to get a drug prescription than city/town/urban area (11.1%), or coastal fishing community (6.2%). A significant relationship was found between education levels and drug prescriptions, [X^2^ (6, *N* = 451) = 27.977, *p* < 0.001]. Participants with education levels between Grade 6–O/Ls (36.4%) were more likely to get a drug prescription than those with no school education (6.3%), Grade 1–5 (31.3%) or passed their O/Ls (15.2%). Similarly, the relation between occupation and drug prescription at an OP service was significant, [*p* = 0.002], with those employed (41.4%) or at home/housewife (43.8%) more likely to get a drug prescription than those unemployed/off sick/disabled (10.3%), or a student (2.2%) (Table 6).

112 (10.9%) of Participants stated they attended op services for a follow-up appointment. A significant relationship was found between age groups and follow-up appointments at an OP Service, [X^2^ (3, *N* = 112) = 24.984, *p* < 0.001], with those aged 50–64 (42.9%) more likely to visit an OP Service for a follow-up appointment than participants aged 18–34 (3.6%), 35–49 (16.1%) or 65 + (37.5%). The Relationship between gender and follow-up appointment was close to significant, [X^2^ (1, *N* = 112) = 4.313, *p* = 0.038], with female participants (61.6%) slightly more likely to attend OP Services for follow-up appointments than male participants (38.4%) (Table 6).

A binary logistic regression (Table 7) notes significant associations between outpatients seen for general diagnoses and education below O/L [OR, 2.021; 95% CI, 1.52–2.67; *p* > 0.000]. Table 8 notes a significant association between outpatients seen for follow-up appointments and age above 50 years [OR, 3.044; 95% CI, 1.87–4.94, *p* > 0.000].

**Table 7:** Binary logistic regression with outpatients seen for general diagnoses.

***n* = 1,019**	**Adj odds ratio**	***P*-value**	**95.0% CI**	
			**Lower**	**Upper**
**Education**				
Above O/L 278 (27.3%)	1 (Ref)			
Below O/L 741 (72.7%)	2.021	**0.000**	1.525	2.678
**Location of home**				
Urban/city 89 (9.7%)	1 (Ref)			
Rural/coastal area 930 (91.3%)	0.416	**0.000**	0.264	0.656

*Negelkerke R^2^ = 0.053, Diagnosis; yes = 586, no = 433. Bold values are statistically significant results*.

**Table 8:** Binary logistic regression with outpatients seen for follow-ups.

***n* = 1,019**	**Adj odds ratio**	***P*-value**	**95.0% CI**	
			**Lower**	**Upper**
**Age**				
Below 50 years 416 (40.8%)	1 (Ref)			
Above 50 years 603 (59.2%)	3.044	**0.000**	1.873	4.948
**Employment**				
Currently employed 436 (42.8%)	1 (Ref)			
Unemployed/ student/retired 583 (57.2%)	1.468	0.073	0.964	2.235

*Negelkerke R^2^ = 0.056, Follow-up; yes = 112, no = 907. Bold values are statistically significant results*.

#### Where Outpatient Treatment Was Sought and Healthcare Professional Seen

Out of the 1,019 participants, 999 (98.0%) indicated they sought treatment at a divisional hospital rather than a primary medical care unit and the majority of participants indicated they saw a general medical doctor (Table 9). Table 9 notes a significant relationship found between home location and visits to a mental health professional, [*p* < 0.001], with over three quarter of participants from a village/rural area (80.3%) more likely to visit a mental health professional than participants from a city/town/urban area (16.8%), plantation (2.3%) or coastal fishing community (0.6%). A significant relationship was found between age groups and visits to a traditional healer, [X^2^ (3, *N* = 317) = 18.328, *p* < 0.001], with both 50–64 (33.8%) and 65+ (33.8%) age groups just as likely to visit a traditional healer, but 35–49 (22.7%) and 18–34 (9.8%) age groups less likely to visit a traditional healer (Table 9).

**Table 9:** Outpatient and healthcare provider seen.

	**General MD**	**Mental health professional**	**Traditional practitioner**
**Age groups**			
18–34	120 (12.9%)	34 (19.7%)	31 (9.8%)
35–49	248 (26.6%)	52 (30.1%)	72 (22.7%)
50–64	313 (33.5%)	51 (29.5%)	107 (33.8%)
65+	252 (27%)	36 (20.8%)	107 (33.8%)
Pearson chi-squared	0.006	**0.032**	**0.001**
**Ethnicity**			
Tamil	834 (89.4%)	168 (97.1%)	295 (93.1%)
Muslim	99 (10.6%)	5 (2.9%)	22 (6.9%)
Pearson chi- square	0.508	**0.001**	**0.012**
**Gender**			
Male	443 (47.5%)	90 (52%)	150 (47.3%)
Female	490 (52.5%)	83 (48%)	167 (52.7%)
Pearson chi-squared	0.894	0.204	0.908
**Location**			
Rural/village/plantation	851 (91.4%)	144 (83.2%)	287 (90.5%)
Urban/city/town	80 (8.6%)	29 (16.8%)	30 (9.5%)
Pearson chi-squared	-	-	-
Fisher's exact test	**0.001**	**0.001**	0.735
**Occupation**			
Employed	397 (44.9%)	89 (52.0%)	119 (39.4%)
Unemployed/off sick/disabled	96 (10.9%)	17 (10.0%)	41 (13.6%)
Student	13 (1.5%)	3 (1.7%)	5 (1.7%)
Housewife/at home	377 (42.7%)	62 (36.3%)	137 (45.3%)
Pearson chi-squared	0.062	**0.034**	0.122
**Education**			
Not had school education	66 (8.0%)	5 (3.3%)	16 (5.9%)
From grade 1–grade 5	285 (34.5%)	52 (34.0%)	94 (35.0%)
From grade 6–O/Ls	347 (41.9%)	61 (39.8%)	120 (44.4%)
Passed O/Ls	129 (15.6%)	35 (22.9%)	40 (14.8%)
Pearson chi-squared	-	-	-
Fisher's exact test	**0.001**	**0.026**	0.500

*Bold values are statistically significant results*.

The binary logistic regression (Table 10) found a weak association between outpatients who saw a healthcare provider or mental health professional and older age [OR, 0.648; 95% CI, 0.46–0.90; *p* > 0.012], and a significant association with Tamil ethnicity [OR, 4.09; 95% CI, 1.95–12.28, *p* > 0.001]. Table 11 indicates a significant association between outpatients who saw a healthcare provider or traditional practitioners and older age [OR, 1.642; 95% CI, 1.24–2.17, *p* > 0.001], and Tamil ethnicity [OR, 1.944; 95% CI, 1.19–3.17, p > 008].

**Table 10:** Binary logistic regression with outpatients who saw a health care provider or mental health professional.

***n* = 1,019**	**Adj odds ratio**	***P*-value**	**95.0% CI**	
			**Lower**	**Upper**
**Age**				
Below 50 years 416 (40.8%)	1 (Ref)			
Above 50 years 603 (59.2%)	0.648	**0.012**	0.463	0.908
**Ethnicity**				
Muslim 110 (10.8%)	1 (Ref)			
Tamil 909 (89.2%)	4.091	**0.001**	1.955	12.284
**Employment**				
Currently employed 436 (42.8%)	1 (Ref)			
Unemployed/ student/retired 583 (57.2%)	0.634	0.080	0.452	0.889
**Location of home**				
Urban/city 89 (9.7%)	1 (Ref)			
Rural/coastal area 930 (91.3%)	2.945	0.000	1.800	4.818

*Negelkerke R^2^ = 0.075, Mental health professional; yes = 173, no = 846. Bold values are statistically significant results*.

**Table 11:** Binary logistic regression with outpatients who saw a health care provider or traditional practitioner.

***n* = 1,019**	**Adj odds ratio**	***P*-value**	**95.0% CI**	
			**Lower**	**Upper**
**Age**				
Below 50 years	1 (Ref)			
Above 50 years	1.642	**0.001**	1.240	2.174
**Ethnicity**				
Muslim	1 (Ref)			
Tamil	1.944	**0.008**	1.190	3.174
**Employment**				
Currently employed	1 (Ref)			
Unemployed/ student/retired	1.314	0.052	0.998	1.730

*Negelkerke R^2^ = 0.034, Traditional practitioner; yes = 317, no = 702. Bold values are statistically significant results*.

#### Outpatient Fees Paid

A total of 161 participants reported paying fees at outpatient services (Table 12). Participants from rural areas appeared most likely to pay fees for OP services, with 44 (88.0%) indicating they paid between 101 and 500 Sri Lankan rupees (0.50–2.50 USD) out of pocket. There was a marginally significant association between any fees paid and home location [X^2^ = (10, *N* = 161) = 27.551, *p* < 0.046].

**Table 12:** Outpatient fee expenditure.

**(*N* = 161)**	**Fees paid:** **under 100**	**Fees paid:** **101–500**	**Fees paid:** **501–1000**	**Fees paid:** **Over 1,000**	**Any fees paid**
**Location of home**					
Rural/village/plantation/coastal	94 (95.9%)	44 (88.0%)	2 (100.0%)	5 (50.0%)	
Urban/city/town	4 (4.1%)	6 (12.0%)	0 (0.0%)	5 (50.0%)	
Fisher's exact test	-	-	-	-	**0.046**

*Bold values are statistically significant results*.

### Inpatient Characteristics

A total of 378 (37%) participants stayed overnight at an inpatient facility (Table 13). The association between occupation and an overnight stay at a hospital was significant, [p < 0.001], with participants in employment (43.9%) more likely to stay overnight at a hospital than participants who were at home/housewife (36.2%) or unemployed/off sick/disabled (13%). A significant association found with male participants (67.8%) more likely to seek inpatient treatment for injuries than females (32.2%), [X^2^ (1, N = 115) = 18.019, *p* < 0.001] (Table 13).

**Table 13:** Inpatient admission and reason for visit.

	**Overnight stay over past 12 months**	**Injury**	**Chronic disease**	**Other reason**
**Age groups**				
18–34	38 (10.1%)	15 (13%)	1 (1.2%)	14 (7.1%)
35–49	82 (21.7%)	25 (21.7%)	10 (12%)	43 (21.8%)
50–64	139 (36.8%)	33 (28.7%)	38 (45.8%)	78 (39.6%)
65+	119 (31.5%)	42 (36.5%)	34 (41%)	62 (31.5%)
Pearson chi-squared	**0.001**	0.125	**0.001**	0.217
**Ethnicity**				
Tamil	335 (88.6%)	111 (96.5%)	64 (77.1%)	177 (89.8%)
Muslim	43 (11.4%)	4 (3.5%)	19 (22.9%)	20 (10.2%)
Pearson chi-squared	**0.652**	**0.001**	**0.001**	0.435
**Gender**				
Male	194 (51.3%)	78 (67.8%)	34 (41%)	89 (45.2%)
Female	184 (48.7%)	37 (32.2%)	49 (59%)	108 (54.8%)
Pearson chi-squared	**0.064**	**0.001**	**0.033**	**0.013**
Fisher's exact test		n/a	n/a	n/a
**Occupation**				
Employed	166 (44.4%)	56 (48.7%)	23 (27.7%)	81 (41.1%)
Unemployed/off sick/disabled	49 (13.1%)	18 (15.7%)	15 (18.1%)	32 (16.2%)
Housewife/at home	137 (36.6%)	33 (28.7%)	39 (47%)	72 (36.5%)
Student	0	1 (0.80%)	0	2 (1.02%)
Retired/other	22 (5.9)	7 (6.1%)	5 (6%)	10 (5.08%)
Fisher's exact test	0.001	0.391	**0.005**	0.274
**Education**				
Up to grade 5	177 (51.7%)	13 (11.3%)	8 (9.6%)	25 (12.7%)
From grade 6–O/Ls	124 (36.3%)	33 (28.7%)	34 (41%)	83 (42.1%)
Passed O/Ls	41 (12.0%)	17 (14.8%)	7 (8.4%)	20 (10.2%)
Fisher's exact test	**0.001**	0.173	0.368	n/a
**House location**				
Rural/village/plantation/coastal	49 (90.7%)	18 (90.0%)	14 (100%)	21 (87.5%)
Urban/city/town	5 (9.3%)	2 (10.0%)	0 (0%)	3 (12.5%)
Fisher's exact test	0.663	0.886	0.221	0.464

*Bold values are statistically significant results*.

The most visited healthcare provider for inpatient services were Divisional Hospitals with a total of 375 participants visiting them, with a total of 3 participants visiting primary care units and 1 participant visiting private services (Table 14).

**Table 14:** Inpatients and choice of healthcare facility.

	**Divisional hospital**	**Primary medical care unit**	**Private health facility**
**Age groups**			
18–34	38 (10.1%)	-	-
35–49	82 (21.9%)	-	-
50–64	138 (36.8%)	2 (66.7%)	-
65+	117 (31.2%)	1 (33.3%)	1 (100%)
Pearson chi-squared	n/a	n/a	n/a
Fisher's exact test	0.420	0.474	0.509
**Ethnicity**			
Tamil	333 (88.8%)	1 (33.3%)	1 (100%)
Muslim	42 (11.2%)	2 (66.7%)	-
Pearson chi-squared	n/a	n/a	n/a
Fisher's exact test	0.312	**0.023**	0.623
**Occupation**			
Employed	166 (44.3%)	1 (33.3%)	-
Unemployed/off sick/disabled	49 (13.1%)	-	-
Housewife/at home	134 (35.7%)	2 (66.7%)	1 (100%)
Retired	22 (5.9%)	-	-
Fisher's exact test	0.294	0.863	0.844
**Education**			
Up to grade 5	133 (35.5%)	2 (66.7%)	-
From grade 6–O/Ls	123 (32.8%)	-	1 (100%)
Passed O/Ls	41 (10.9%)	-	-
Pearson chi-squared	n/a	n/a	n/a
Fisher's exact test	0.681	0.360	0.897
**Location**			
Rural/village/plantation	328 (86.1%)	2 (66.7%)	-
Urban/city/town	28 (7.5%)	1 (33.3%)	1 (100%)
Coastal fishing community	19 (5.1%)	-	-
Pearson chi-squared	n/a	n/a	n/a
Fisher's exact test	**0.035**	0.401	0.078

*Bold values are statistically significant results*.

A binary logistic regression (Table 15) notes significant associations between inpatients admitted for chronic disease and older age [OR, 4.857; 95% CI, 2.52–9.33, *p* > 0.000], Tamil ethnicity [OR, 0.340; 95% CI, 0.19–0.60, *p* > 0.000], and a marginal association with being unemployed/student/retired [OR, 1.875; 95% CI, 1.12–3.12, *p* > 0.016]. Table 15 also notes significant associations between those admitted overnight and being over 50 years of age [OR, 1.676; 95% CI, 1.27–2.2, *p* > 0.000], and a marginal association in those with lower education levels [OR, 1.542; 95% CI, 1.12–2.10, *p* > 0.007].

**Table 15:** Binary logistic regression with inpatients.

**Admitted for chronic disease (*n* = 1,019)**	**Adj odds ratio**	***P*-value**	**95.0% CI**	
			**Lower**	**Upper**
**Age**				
Below 50 years 416 (40.8%)	1 (Ref)			
Above 50 years 603 (59.2%)	4.857	**0.000**	2.528	9.334
**Ethnicity**				
Muslim 110 (10.8%)	1 (Ref)			
Tamil 909 (89.2%)	0.340	**0.000**	0.191	0.605
**Employment**				
Currently employed 436 (42.8%)	1 (Ref)			
Unemployed /student/retired 583 (57.2%)	1.875	**0.016**	1.126	3.120
**Location of home**				
Urban/city 89 (9.7%)	1 (Ref)			
Rural/coastal area 930 (91.3%)	1.612	0.180	0.802	3.238
**Admitted overnight** (*n* = 1,019)				
Age				
Below 50 years 416 (40.8%)	1 (Ref)			
Above 50 years 603 (59.2%)	1.676	**0.000**	1.272	2.209
**Education**				
Above O/L 278 (27.3%)	1 (Ref)			
Below O/L 741 (72.7%)	1.542	**0.007**	1.128	2.108

*Negelkerke R^2^ = 0.105, Chronic disease; yes = 205; no = 814, Negelkerke R^2^ = 0.038, Overnight admission; yes = 379; no = 640. Bold values are statistically significant results*.

## Discussion

Sri Lanka's healthcare system was designed to provide universal healthcare services to all citizens and operates in a robust fashion despite almost 30 years of conflict. The Northern Province was particularly affected by the conflict and is still recovering. Data from this region on utilization of healthcare services is sparse but essential to understand to ensure allocation of healthcare expenditure and resources is completed effectively. The results from this study indicate the majority of those seeking healthcare services in Northern Province are from rural areas, are older adults (50 years +), are of Tamil ethnicity, have lower education levels, and are unemployed/retired or students. The majority of participants who sought outpatient services attended divisional hospitals and sought help for diagnoses and follow-up. A significant number of outpatient participants sought care with mental health professionals and traditional practitioners. Importantly, the majority of those who sought outpatient services stated they paid some sort of fee at point of contact, which is in contradiction to the country's universal health care design. For patients who received inpatient care (only available at divisional hospitals), the majority were over 50 years, of Tamil ethnicity, had lower education levels, were located in rural or coastal areas, and had lower educational levels. Inpatients appeared to attend facilities for chronic diseases, acute diseases and injuries. The results indicate a pattern of service utilization that is heavily focused on divisional hospitals as participants appear to have a preference over local primary medical care units, which provide outpatient care in each district. This could be due to hours of operation (divisional hospitals tend to be 24 h, while primary medical care units often close in the late afternoon), lack of services, supplies, or human resources. While primary medical care units were created to take pressure of the larger, and more scare, divisional hospitals, our results indicate they are underutilized. Findings from this study have been grouped and interpreted using three thematic areas: health education, health resource allocation, and healthcare fees.

### Health Education

There was a significant association between those who were over 50 years, of Tamil ethnicity, unemployed, retired or students, and living in rural and coastal areas and use of outpatient services. Importantly, the majority of participants appeared to favor urban divisional hospitals over local primary medical care units when seeking out outpatient care. Outpatient services were most often sought for diagnoses and follow-up this suggests the need for education on outpatient services available at local primary medical care units. Results also suggest a majority of participants living in rural area sought help for acute conditions in outpatient services, and those seeking help for sleep problems also originate in rural areas. However, the majority of those seeking care bypassed local outpatient-only primary medical care units to seek care at larger, urban divisional hospitals. This is similar to work done by Lee et al. (11) who noted in their work in post conflict Liberia that a significant rural health delivery gap. However, in Sri Lanka's case this rural health delivery gap does not seem to be associated with lack of facilities, but preference for larger, urban centered divisional hospitals.

Further, it was noted that more than half of participants with an education of Grade 6 onwards were more likely to seek outpatient treatment for acute conditions than those with less education. Again, this indicates the need for health education programs to share messages around injury and acute conditions, particularly in rural areas. This is supported by work done by the Ministry of Health, Nutrition, and Indigenous Medicine which notes the need to create programs at community level that address lifestyle changes (10). Health education has been a valuable tool to better equip people to address their health issues and an essential component of action to promote population health (19). Health is significantly determined by diverse social, economic, and environmental characteristics of individuals. This is particularly salient to this study as it appears that there is an unmet need for health education that is sensitive to local experiences of conflict, education and income levels.

Health messaging has, in the past, been composed of simple transmission of information. However, over time it was understood that simple message transmission is not enough, health education must take into account social and economic circumstances of the target audience (19). This is especially important in post conflict settings as social and economic circumstances may have been significantly disrupted and require thoughtful messaging to ensure messages are received and accepted. In Sri Lanka's setting, it appears to also be important to include messaging on where to access services more appropriately to take strain off urban divisional centers. As an estimated 75% of death in the country can be attributed to NCDs such as cardiovascular disease, stroke, cancer, and diabetes (8, 10), health messaging must not only educate people on NCDs, but also where to seek care in appropriate ways to ensure healthcare pathways are used effectively. This is supported by our findings which note significant associations between those over 50 years of age and seeking inpatient services for chronic disease. With a growing population of those seeking care for chronic disease, investment in health messaging will be required to ensure chronic disease risks are reduced as much as possible. Non-communicable and chronic diseases are on the rise in Sri Lanka along with other developing countries and this can greatly increase the burden on healthcare systems (20). This is supported by research done in Albania that noted nearly half their participant population were older adults (60+) who attended for chronic illnesses (21).

Boutayeb and Boutayeb (20) indicate health messaging to ensure people are attending appropriate facilities requires investment in messaging around risk factors such as alcohol and tobacco use, physical activity and diet. Further, an article from Mossman et al. (21) recommends expanding the reach of primary care by utilizing business and marketing strategies used by private healthcare. They note using the tactic of market focus to create relationships with patients who may be bypassing public care due to lack of access to quality care. The authors note the need for more effective market and engagement in health education campaigns to promote available services and focus on patient experience (21). This could be helpful in the case of Sri Lanka, and other similar settings, as a market focus perspective could increase quality of services at localized facilities and in turn, increase uptake of these facilities to decrease strain on larger, urban hospitals.

### Health Resource Allocation

This study found that regardless of whether inpatient or outpatient care was sought, the majority of participants attended divisional hospitals, rather than primary medical care units. Patterns of service utilization suggest that older age groups (50+) are attending for diagnosis and follow-up appointments at outpatient services. Within rural areas it appears there is utilization of primary medical care units for diagnosis, assessment and drug prescriptions, and findings suggest women tend to attend follow-up visits more than men; this could be due to primary medical care units being open during working hours. Further, participants appeared to seek out treatment with mental health professionals and traditional practitioners. As specialized mental health professionals, such as psychiatrists are only located at divisional hospitals (3), this indicates a need to ensure preferred healthcare professionals are available and accessible at local primary medical care units to reduce strain on urban locations. The larger parent study, COMGAP-S, tried to address this gap through training primary care practitioners at primary medical care units to deliver basic mental health services to increase access (15).

In Sri Lanka, divisional hospitals provide inpatient services (hospitalization) and ambulatory care, while primary medical care units provide only ambulatory care and function with non-specialist medical doctors and support staff (20). This indicates that perhaps people are by-passing local primary medical care units for the more specialized divisional hospital environments within larger urban centers. This could possibly be occurring as there is a lack of formalized care pathways so people make decisions to seek out specialized services on their own. Or it could be due to lack of staff, equipment and medicines on site at primary medical care units leading people to seek out the larger divisional hospitals. This is supported by the 2018 work from the Ministry of Health, Nutrition, and Indigenous Medicine which notes the need to expand both services offered (including NCD screening) and expanded service hours to encourage more use of primary medical care units (10). This report also indicates the strong need for clear referral pathways and coordination between services to increase efficient utilization of resources (10).

Future research is needed to understand more clearly why the majority of participants attend divisional hospitals. Understanding the reasons behind participant choices could facilitate better allocation of both financing and human resources and reduce burden on divisional hospitals (21). Perhaps primary medical care units are not easily accessible, perhaps medications and equipment are not always equally distributed across facilities, or perhaps there are staffing issues. This is supported by the Ministry of Health, Nutrition, and Indigenous Medicine's report (10) and by work by Kumar (2) who notes that underinvestment has resulted in widespread shortages of medical supplies, inequitable service distribution and shortages in human resources. Kruk and Freedman (22) note the need to investigate patient satisfaction and access to and quality or care to assess and strengthen health systems in developing countries, and this is also supported by work from Mossman et al. (21) While healthcare professionals are required to serve in remote areas to gain future promotion, there are regional disparities and a constantly moving workforce which could undermine continuity of care. In their work in post conflict Liberia, Lee et al. (11) note a rural healthcare delivery gap along with the need for more effective financing mechanisms to ensure equity of care indicating some similar issues to post conflict Sri Lanka.

Gabrani et al. (23) further note the need to expand and develop primary care facilities through engagement of local decision-makers and other stakeholders. They also note that in their work in Albania it was discovered that participants make choices about primary care facilities based on geography and financial access (23). This is in apparent contradiction with what this current study found as our participants indicated they bypassed local primary medical care units to attend urban centered divisional hospitals. This would also mean increased financial strain due to travel costs, time off work, childcare and other expenses. Future research will need to investigate why participants are bypassing the decentralized system of primary medical care units in favor of divisional hospitals.

Further, when healthcare systems are decentralized, for example the creation of primary medical care units in local areas to reduce strain on divisional hospitals, there needs to be a performance assessment or evaluation performed. This is supported by Lee et al. (11) in post conflict Liberia, who note the need for efficient alignment of healthcare infrastructure with existing need. As primary medical care units are underutilized and bypassed for larger urban divisional hospitals, the existing healthcare structure within Sri Lanka either needs to change to adapt to use, or increase messaging to encourage use of structure as it currently exists.

Bossert (24) recommends understanding what kinds of services have been transferred, and what effects that has on the performance of the healthcare system overall. To understand why primary medical care units are being bypassed, this kind of evaluation would be valuable to decision makers within Sri Lanka, and other settings where health systems have been decentralized in a similar manner.

Evidence on healthcare utilization suggests that those with more financial resources tend to seek out private healthcare to avoid the waiting times, inconvenient government operating hours, and limited provider choice (8, 25). If primary medical care units are not being utilized to their capacity, this could increase burden on divisional hospitals. Further, as NCD prevalence increases, it will be more and more essential to ensure proper screening and detection occurs within formalized healthcare service pathways. To counter this, Mossman et al. (21) recommend partnering public and private organizations as ways to increase access to financial support, healthcare staff and medical supplies. This may be feasible in Sri Lanka, along with other developing contexts, through partnerships with some of the many not-for-profits that operate social and healthcare programs. Further, the authors note the importance of training medical leaders in business acumen as this may help provide more high quality and efficient services (21).

With limited healthcare resources and financing to allocate across the entire country, it is essential to understand why some facilities are under- or over-utilized, particularly due to the COVID-19 pandemic. This could also ensure clear pathways to care are formalized for those seeking care enabling services to work more effectively and efficiently. This is a common issue across post conflict settings that need to find solutions to ensure access and parity of care across large regions which may have been significantly disrupted during conflict.

### Healthcare Fees

While Sri Lanka has a publicly funded universal healthcare system, outpatients within this study indicate they paid fees at point-of-contact. This indicates that perhaps Sri Lanka's healthcare system does not yet fully meet the World Health Organization's definition of universal healthcare. The most commonly cited reason for out-of-pocket payments in this study was for medications, and this is supported by work done by Wang et al. (14). This is also supported by a community-based research within the country that noted an increase in fee-for-service payments made for healthcare and the author reports this can increased significantly over the past decade (3).

These out-of-pocket payments are concerning as poorer households within the Northern Province could be disproportionately affected as they often have fewer resources and reduced access to resources (14). This is compounded by the fact that Northern Province was heavily affected by the conflict and infrastructure and services are still recovering. Out-of-pocket expenditure can create financial hardship, which can be conceptualized as (1) catastrophic spending and (2) impoverishment (14). Catastrophic spending impedes the ability to pay for necessities of life, while impoverishment occurs when spending pushes people into poverty. Either of these could negatively impact those living in Northern Province and cause larger issues for the delivery and receipt of healthcare. If healthcare costs begin to cause financial hardship, people will limit their contact with healthcare increasing their risk of presenting with chronic or late-stage diseases (26). This is especially salient in a country such as Sri Lanka which is seeing increasing numbers of NCDs which require clear pathways of care (8). This is supported by work done in similar post conflict settings which recommend increased investment in healthcare services and delivery to ensure healthcare services are available to all who need them (12). Mossman et al. (21) also note the need for financial strategies to help people access services without payment at point-of-contact and to generate income where resources may be limited. This may mean creation of health plans or micro-insurance plans or membership fees to offset costs of providing care without causing catastrophic spending at patient level.

### Strengths and Limitations

This study was a quantitative data analysis of a larger data set collected for the COMGAP-S study (15). The data collected was largely quantitative so further qualitative investigation into why choices were made were not able to be investigated. It is recommended that future studies qualitatively explore the choices by those seeking healthcare in Northern Province to better understand decisions made. The weakness of this study is that it relied on self-disclosed information from participants. The data could have been impacted by recall bias as memories could have faded or memories were selectively chosen to share by participants. A further weakness is that the parent study, COMGAP-S only concentrated on public primary care facilities, not private facilities. There is no existing list of private healthcare facilities in Sri Lanka, therefore the investigators only included public healthcare facilities that are run by Ministry of Health. As citizens have the option to attend private, for-pay, healthcare sites we could have missed important aspects of healthcare utilization in these facilities. Future research should endeavor to identify and include private facilities to address this. The strength of this study is that it is the first study, that we know of, to investigate the healthcare utilization and characteristics of post-conflict, adult attendees of outpatient and inpatient, publicly funded healthcare services. This study is in line with current work being conducted in Sri Lanka from the World Bank and the Ministry of Health, Nutrition and Indigenous Medicine, and supports current policy proposals regarding re-organizing primary care and achieving pro-poor universal health care coverage (8, 10, 20, 21, 24). It is hoped that lessons learned could be useful to other similar post conflict settings and developing country contexts who may struggle with similar issues.

## Conclusion

This study illuminates the previously unknown patterns of utilization of healthcare services at primary care level and identified characteristics of conflict-affected adults who attended primary care facilities within Northern Province, Sri Lanka. We found that there are significant associations between older age, employment, lower education and living in rural or coastal areas and healthcare utilization among conflict-affected, adult, primary care attendees particularly for chronic disease. This study indicates health seeking preferences, such as utilization of divisional hospitals with specialized services over more locally available primary medical care units. Results indicate primary care medical units are being primarily utilized by older groups, possibly due to limited clinic hours or the inability to travel to urban centers to divisional hospitals. Divisional hospitals appear overutilized perhaps due to access through increased hours or availability of specialist care or increased access to services. Understanding the reasons behind participant choices could facilitate better allocation of both financing and human resources and reduce burden on divisional hospitals. This is especially important due to the strain on healthcare systems due to the COVID-19 pandemic. Further, creating clear referral pathways could increase efficient care pathways within Sri Lanka to ensure resources are used effectively. The findings of this study indicate the need for further investigation into the reasons behind health seeking behaviors to increase efficiency of healthcare resource allocation and effective referral pathways. Additionally, the results of this study indicate the need for a health education program to reduce risky behaviors and improve health seeking behaviors to ensure pathways of care are used effectively and efficiently. Lessons learned from this study could help local decision-makers, and others in similar developing country and post-conflict settings expand effective and efficient access to, and use of, primary health care facilities to ensure needs are met. Globally, there are limited resources available to allocated to primary care services and it is imperative that resources are applied effectively and efficiently, particularly due to the increased pressure of COVID-19.

## Data Availability Statement

The raw data supporting the conclusions of this article will be made available by the authors, without undue reservation.

## Ethics Statement

This study received ethics approval from the Faculty Research Ethics Panel, Faculty of Health, Education, Medicine and Social Care, Anglia Ruskin University, UK (FMSFREP 16/17 076). Ethics approval was also granted from the Ethics Review Committee, Faculty of Medicine, University of Jaffna, Sri Lanka (J/ERC/17/81/NDR/0170). The patients/participants provided their written informed consent to participate in this study.

## Author Contributions

SD, DS, RS, and RT conceived and designed the manuscript. SD and RS wrote the manuscript, RT conducted statistical analyses and assisted with interpretation, while DS created statistical analysis tables and participated in revision of the manuscript. All authors made substantial contributions to conception and design, acquisition of data, or analysis, and interpretation of data, critically revised manuscript, and approved the final version.

## Funding

This publication was supported by the Cooperative Agreement Number GH001654, funded by the Centers for Disease Control and Prevention. This project was also supported by the Northern Ministry of Health, Sri Lanka, and the University of Jaffna. The Funding organization was involved in consultations regarding the validity of the design, and the Northern Ministry of Health and the University of Jaffna were involved in consultations on the design of the study, collection and interpretation of data.

## Author Disclaimer

Its contents are solely the responsibility of the authors and do not necessarily represent the official views of the Centers for Disease Control and Prevention or the Department of Health and Human Services.

## Conflict of Interest

The authors declare that the research was conducted in the absence of any commercial or financial relationships that could be construed as a potential conflict of interest.

## Publisher's Note

All claims expressed in this article are solely those of the authors and do not necessarily represent those of their affiliated organizations, or those of the publisher, the editors and the reviewers. Any product that may be evaluated in this article, or claim that may be made by its manufacturer, is not guaranteed or endorsed by the publisher.

## References

[1] Pallegedara A Grimm M. Demand for private healthcare in a universal public healthcare system: empirical evidence from Sri Lanka. Health Policy Plan. (2017) 32:1267–84. 10.1093/heapol/czx085 28981660

[2] Kumar R. Public–private partnerships for universal health coverage? the future of “free health” in Sri Lanka. Globalization Health. (2019) 15:75. 10.1186/s12992-019-0522-6 31775851 PMC6882304

[3] Mendis K. Healthcare Indicators. (2021). Available online at: https://kmendis.net/2021/08/09/primary-care-family-medicine-and-covid-19-pandemic-in-sri-lanka/ (accessed August 12, 2021).

[4] Sachs JD. From millennium development goals to sustainable development goals. Lancet. (2012) 379:2206–11. 10.1016/S0140-6736(12)60685-0 22682467

[5] UNICEF. Sri Lanka (LKA)–Demographics, Health & Infant Mortality. (2020). Available online at: https://data.unicef.org/country/lka/ (accessed January 19, 2021).

[6] World Bank. Sri Lanka Context. (2019). Available online at: https://www.worldbank.org/en/country/srilanka/overview (accessed January 19, 2021).

[7] World Bank. World Bank Data. (2018). Available online at: https://data.worldbank.org/indicator/SP.DYN.LE00.IN?locations=LK (accessed January 19, 2021).

[8] Perera S Nieveras O de Silva P Wijesundara C Pendse R. WHO South-East Asia Region. Bulletin World Health Organisation. (2019) 8:21–5. 10.4103/2224-3151.255345 30950426

[9] Ministry of Health, Medical Statistics Unit. Annual Health Statistics in Sri Lanka (2019).

[10] Ministry of Health, Nutrition and Indigenous Medicine. Reorganising Primary Health Care in Sri Lanka: Preserving Our Progress, Preparing Our Future (2018).

[11] Lee PT Kruse GR Chan BT Massaquoi MB Panjabi RR Dahn BT. An analysis of Liberia's 2007 national health policy: lessons for health systems strengthening and chronic disease care in poor, post-conflict countries. Global Health. (2011) 7:1–4. 10.1186/1744-8603-7-37 21985150 PMC3201890

[12] Nelson BD Simic S Beste L Vukovic D Bjegovic V VanRooyen MJ. Multimodal assessment of the primary healthcare system of Serbia: a model for evaluating post-conflict health systems. Prehosp Disaster Med. (2003) 18:6–13. 10.1017/S1049023X00000613 14694894

[13] Kentoffio K Kraemer JD Griffiths T Kenny A Panjabi R Sechler GA. Charting health system reconstruction in post-war Liberia: a comparison of rural vs. remote healthcare utilization. BMC Health Serv Res. (2016) 16:1–9. 10.1186/s12913-016-1709-7 27604708 PMC5015243

[14] Wang H Vinyals Torres L Travis P. Financial protection analysis in eight countries in the WHO South-East Asia Region. Bull World Health Organ. (2018) 96:E610–20. 10.2471/BLT.18.209858. 10.2471/BLT.18.209858 30262942 PMC6154066

[15] Doherty S Hulland E Lopes-Cardozo B Kirupakaran S Surenthirakumaran R Cookson S. Prevalence of mental disorders and epidemiological associations in post-conflict primary care attendees: a cross-sectional study in the Northern Province of Sri Lanka. BMC psychiatry. (2019) 19:83. 10.1186/s12888-019-2064-0 30832646 PMC6399832

[16] Husain F Anderson M Cardozo BL Becknell K Blanton C Araki D. Prevalence of war-related mental health conditions and associations with displacement status in postwar Jaffna District, Sri Lanka. JAMA. (2011) 306:522–31. 10.1001/jama.2011.1052 21813430

[17] Siriwardhana C Adikari A Pannala G Siribaddana S Abas M Sumathipala A. Prolonged internal displacement and common mental disorders in Sri Lanka: the COMRAID study. PLoS ONE. (2013) 8:e64742. 10.1371/journal.pone.0064742 23717656 PMC3661540

[18] IBM Corp. IBM SPSS Statistics For Windows, Version 26.0. Armonk, NY: IBM Corp (2019).

[19] Nutbeam D. Health literacy as a public health goal: a challenge for contemporary health education and communication strategies into the 21^st^ century. Health Promot Int. (2000) 15:259–67. 10.1093/heapro/15.3.259

[20] Boutayeb A Boutayeb S. The burden of non communicable diseases in developing countries. Int J Equity Health. (2005) 4:1–8. 10.1186/1475-9276-4-2 15651987 PMC546417

[21] Mossman K Bhattacharyya O McGahan A Mitchell W. Expanding The Reach Of Primary Care In Developing Countries. Harvard Business Review. (2017). Available online at: https://hbr.org/2017/06/expanding-the-reach-of-primary-care-in-developing-countries (accessed August 9, 2021).

[22] Kruk ME Freedman LP. Assessing health system performance in developing countries: a review of the literature. Health Policy. (2008) 85:263–76. 10.1016/j.healthpol.2007.09.003 17931736

[23] Gabrani J Schindler C Wyss K. Factors associated with the utilisation of primary care services: a cross-sectional study in public and private facilities in Albania. BMJ Open. (2020) 10:e040398. 10.1136/bmjopen-2020-040398 33262191 PMC7709502

[24] Bossert T. Analyzing the decentralization of health systems in developing countries: decision space, innovation and performance. Soc Sci Med. (1998) 47:1513–27. 10.1016/S0277-9536(98)00234-2 9823047

[25] Perera S. Primary health care reforms in Sri Lanka: aiming at preserving universal access to health. In: Medcalf A Bhattacharya S Momen H, editors. Health For All: The Journey of Universal Health Coverage. Hyderabad: Orient Blackswan (2015). Available online at: https://www.ncbi.nlm.nih.gov/books/NBK316262/26378335

[26] Smith O. “Sri Lanka: Achieving pro-poor universal health coverage without health financing reforms.” Universal Health Coverage Study Series No. 38, World Bank Group, Washington.

